# Surgery at the Base

**Published:** 1919

**Authors:** 


### SURGERY AT THE BASE

The Research Committee of the American Red Cross, desiring a record of the experiences of the United States Army Surgeons in the hospitals in France, decided to devote one session of the Research Society to a Conference on Surgery at the Base. A questionnaire was prepared by Brigadier-General J. M. T. Finney and Colonel George W. Crile to be sent to the various Base Hospitals to get staff replies for use as a basis for discussion. The questionnaire was quite comprehensive in that it dealt with a number of phases of some of the most important hospital problems, particularly those related to war wounds.

This questionnaire on Surgery at the Base was sent to the Commanding Officers of various hospitals with a request for replies expressing the opinions and giving the clinical experience of the surgical staffs. The various hospitals replied promptly, and in a way which showed their interest in their work. Many more replies came to hand than were anticipated, and the discussions on a number of problems, although necessarily brief, are a distinct contribution to the surgical literature of the war. The manuscripts are on file in the Department of Research and Intelligence, 2, Place de Rivoli, Paris, and later will be sent to be kept with the Archives of the Red Cross at Washington.

Since the meetings of the Research Society have been discontinued, it has been decided to publish a summary of the replies, giving the statistics of a number of hospitals as to admissions, number of deaths, etc., and the concensus of opinion expressed by the replies received from the various hospital staffs. Major T. W. Burnett, temporarily on duty at American Military Hospital No. 2, was kind enough to compile this data, which appears in this number of *War Medicine.*

There was a divergence of opinion regarding a number of surgical and hospital problems mentioned in the questionnaire, so that it was difficult, if not impossible, to form composite answers to the questions under consideration. Besides, many of the replies were considered of such value that it was decided to select ten representative replies — not the ten best, because with so much excellent material at hand it would not be possible to decide which responses could be considered the best — and to publish these in full in order that those interested might have a better conception of the surgical technic employed and the work accomplished by the surgeons in American hospitals in France. It is to be regretted that all the replies could not be published in full. The material, however, would fill a large volume, and publishing such a book would be an undertaking that could not be carried out at this time.

This record of surgical accomplishment in American hospitals in France will not only be part of surgical history, and of value for use in the future should ever the necessity arise, but surgeons will find much in this summary and recital of the experiences of many of their confreres that will be helpful to them when they return to their work at home.

